# Comparative Analysis of Mutation Status and Immune Landscape for Squamous Cell Carcinomas at Different Anatomical sites

**DOI:** 10.3389/fimmu.2022.947712

**Published:** 2022-07-22

**Authors:** Wenqi Ti, Tianhui Wei, Jianbo Wang, Yufeng Cheng

**Affiliations:** Department of Radiation Oncology, Qilu Hospital, Cheeloo College of Medicine, Shandong University, Jinan, China

**Keywords:** squamous cell carcinoma, immune cell infiltration, tumor mutation burden, prognosis, the Cancer Genome Atlas database

## Abstract

**Objective:**

It has been controversial whether tumor mutation burden (TMB) affects the prognosis and the efficacy of immunotherapy in different tumor types. We provided a comprehensive analysis of mutation status and immune landscape of squamous cell carcinomas (SCCs) from four sites in order to investigate the relationship of TMB with prognosis and immune cell infiltration in different SCCs.

**Methods:**

The transcriptome profiles and somatic mutation data of SCCs downloaded from the Cancer Genome Atlas (the Cancer Genome Atlas) database were analyzed and visualized. Then, TMB was calculated to analyze its correlations with prognosis and clinical features. Differentially expressed genes (DEGs) between the high and low TMB groups were screened for functional enrichment analysis. CIBERSORT algorithm was used to compare differences of immune cell infiltration between two groups in different SCCs. In addition, immune DEGs associated with prognosis were identified and risk prediction model was constructed *via* Cox regression analysis.

**Results:**

Missense mutation was the most dominant mutation type in SCCs. The difference was that the top10 mutated genes varied widely among different SCCs. High TMB group had better prognosis in lung squamous cell carcinoma (LUSC) and cervical squamous cell carcinoma (CESC), while the result was reverse in head and neck squamous cell carcinoma (HNSCC) and esophageal squamous cell carcinoma (ESCC). In addition, patients with older age, smoking history, earlier pathological stage and no lymphatic invasion had higher TMB. The identified DEGs were mainly enriched in the regulation of immune system, muscular system and the activity of epidermal cells. The proportions of CD8+T cells, CD4+ memory T cells, follicular helper T cells, macrophages were distinct between two groups. The prognosis-related hub genes (CHGB, INHBA, LCN1 and VEGFC) screened were associated with poor prognosis.

**Conclusion:**

This study reveals the mutation status and immune cell infiltration of SCCs at different anatomical sites. TMB is closely related to the prognosis of SCCs, and its effects on prognosis are diverse in different SCCs, which might result from the situation of immune cell infiltration. These findings contribute to the exploration of biomarkers for predicting the efficacy of immunotherapy in SCCs and providing innovative insights for accurate application of immunotherapy.

## Introduction

Squamous cell carcinomas (SCCs) are the most common human solid tumors, arising from the epithelia of the aerodigestive and genitourinary tracts. They are frequently found in nasal cavity, oropharynx, esophagus, lung and anogenital region (1). SCCs from different parts of the body share some important properties due to their common histopathological features. They share certain common risk factors, such as smoking, excess alcohol drinking and human papillomavirus (HPV) infection (2). Cancer-related DNA hypermethylation is influenced by cell-type-specific chromatin markers or transcriptional programs, resulting in a tendency for some tumors from the same origin to aggregate common methylation data. For example, SCCs (HNSCC, ESCC, LUSC, and CESC) are strongly associated with METH2 and METH3 (3). Therefore, SCCs from different anatomical sites may have similar molecular patterns and clinical outcomes.

Chemotherapy, radiotherapy and surgery are traditional tumor treatments, which often have little effect on some metastatic, recurrent and refractory diseases (4). In recent years, studies of the anti-tumor immune response have promoted the development of therapeutic strategies, among which immunotherapy has become spotlighted in the field of cancer. Immunotherapy regulates the immune system to improve anti-tumor immune response and overcome immune escape mechanism. Because of its high clinical safety, lasting efficacy and effective improvement of survival, it has brought revolutionary innovation and has gradually become the pillar of modern cancer treatment. However, the benefits of immunotherapy remain unclear with low response rates and only a minority of people benefiting from it. A retrospective study using publicly reported cancer statistics and analyses of response rates to immune checkpoint inhibitors (ICIs) treatment found that less than 13% of patients who met immunotherapy indications responded to ICIs (5). Even among melanoma patients with the highest ICIs response rate, the rate was only 40% (6). Immunotherapy bring hope to patients, but also face many challenges in clinical application.

Since immunotherapy is still unable to achieve effectiveness for most cancer patients, there is still a huge space for development of immunotherapy in the era of precision therapy. Therefore, it is necessary to accurately screen potential beneficiaries by predictive biomarkers in order to guide the rational use of therapy in clinical practice. Cancer arises on account of accumulation of somatic mutations and other genetic changes that cause abnormal cell proliferation and ultimately tumorigenesis. With the advancement of high-throughput sequencing, we obtain detailed understanding of the cancer genome and mutational signatures. Most cancers carry between 1,000 and 20,000 somatic mutation, with few to hundreds of insertions, deletions, and rearrangements (7). Tumors induced by exposure to mutagens, such as lung cancer (tobacco) or skin cancer (ultraviolet), tend to have increased mutation rates (8, 9). With regards to this, tumor mutation load (TMB) is used to measure the degree of genetic variation in tumors. TMB is defined as the number of somatic gene non-synonymous mutations in a specific genomic region, which is generally expressed as mutations per million bases (Mut/Mb). Many explorations have revealed that higher nonsynonymous mutation may produce more neoantigens on the surface of tumor cells. These neoantigens can be detected and targeted by the immune system, triggering anti-tumor immune responses and improving the sensitivity of immunotherapy (10). Therefore, as a new biomarker, TMB has been paid more and more attention in predicting the response and prognosis of immunotherapy. In fact, TMB has been shown to be significantly associated with objective response rate to PD-1/PD-L1 inhibitors in a variety of tumors (11). SCC is one of the cancer types with the highest proportion of somatic gene mutations and HLA gene mutations (12). However, the correlation between TMB and the immune landscape in different SCCs has not been systematically studied.

In this study, we explored the mutated genomic pattern and immune cell infiltration in different SCCs. It helps explain the immune escape and limited immunotherapy response rates in SCCs, provides critical insights into common cancer-related genes and regulatory pathways across multiple anatomical sites. This is essential for the widespread use of immunotherapy in solid malignancies.

## Methods

### Data Acquisition and Processing

Clinical information, transcriptome profiles and somatic mutation data of SCC were downloaded from the the Cancer Genome Atlas database (https://portal.gdc.cancer.gov), which is publicly available. We mainly discussed the following four types of SCCs: head and neck squamous cell carcinoma (HNSCC), esophageal squamous cell carcinoma (ESCC), lung squamous cell carcinoma (LUSC), and cervical squamous cell carcinoma (CESC). Clinical data was composed of age, sex, race, smoking history, human papillomavirus (HPV) infection, AJCC-TNM stages, survival time and survival status, etc. RNA-seq data were downloaded in “HTSeq-FPKM” workflow type. The mutation analysis in the Cancer Genome Atlas category “Masked Somatic Mutation” were based on the VarScan program. We visualized the somatic mutation data using “maftools” R package, which commonly provided specific functionality in cancer genomic research.

### TMB Calculation and Evaluation

TMB refers to the total amount of somatic gene coding errors, base substitutions, insertions, or deletions detected per million bases. We calculated TMB as the number of nonsynonymous somatic mutations divide by the length of exons *via* Perl scripts and classified SCC samples into high and low TMB groups based on quartile TMB. Subsequently, we combined the TMB scores with the clinical data. The survival differences between low and high TMB categories were compared using Kaplan-Meier survival analysis and the log-rank test. Wilcoxon rank-sum test and Kruskal-Wallis test were used to analyze the differences of two TMB groups among different clinical traits.

### Differentially Expressed Genes and Functional Enrichment Pathways

Differentially expressed genes (DEGs) were screened by “limma” package between two groups of TMB in SCC, where FDR (false discovery rate) <0.05, and |log2FC (fold change) | >1 were adopted. The heatmap was generated by “pheatmap” package. Then, the Gene Ontology (GO) and Kyoto Encyclopedia of Genes and Genomes (KEGG) pathway analysis of DEGs were displayed with “ggplot2”, “clusterProfiler” and “enrichplot” R packages. Both p-value < 0.05 and q-value < 0.05 were considered as significantly enrichment pathways.

### Estimation of Immune Cell Infiltration

CIBERSORT algorithm was used to evaluate immune cell infiltrations of each sample in the Cancer Genome Atlas database. CIBERSORT, which identifies cell types by estimating relative subsets of RNA transcripts, can accurately calculate the relative content of 22 immune cell from complex tissues. Wilcoxon rank sum test was used to compare the differences of immune cell infiltration between high and low TMB groups in different squamous cells, finally shown in violin plot. When p < 0.05, the results of immune cell fraction inferred by CIBERSORT were statistical significance.

### Identification of Immune-Related DEGs and Construction of Prognostic Model

Immune-related genes were obtained from the immune omics database (https://www.immport.org), and were found the intersection with DEGs through “VennDiagram” package. Overlapping genes were known as immune-related DEGs. Univariate and multivariate Cox regression analysis were used to identify prognostic immune-related DEGs to construct prognostic models by “survival” R package. Risk ratios (HR) and 95% confidence intervals (95% CI) for hub genes in the prognostic model were calculated. In addition, Kaplan-Meier survival analysis and log-rank test were taken to examine the differential survival between high and low expression groups of prognostic immune-related DEGs. P-value < 0.05 was considered with prognostic value. Subsequently, the risk score for each SCC patient was computed based on the prognostic model. The formula was as follows: risk score =Σ (βi×EXPi), where βi stemmed from the multivariate Cox analysis and EXPi represents the expression level of selected immune gene. According to median risk score, patients were divided into low-risk and high-risk groups. Survival differences between the above two groups were compared *via* Kaplan-Meier survival analysis and log-rank test. Finally, the Receiver Operating Characteristic (ROC) curve was performed to assess the predictive value of the constructed prognostic model.

### Relationship Between Prognosis-Related Hub Genes Mutation and Immune Cell Infiltration

We assessed the relationship between the hub genes copy number alteration (CNA) and the level of immune cell infiltration through tumor immune estimation resource (TIMER) database “SCNA” module (https://cistrome.shinyapps.io/timer/). The Somatic Copy Number Alterations (SCNA) module contains the following four CNA: deep deletion, arm-level deletion, arm-level gain, and high amplification. P< 0.05 was considered significant.

### Statistical Analysis

Overall survival (OS) refers to the time interval from the date of diagnosis to the date of death. Survival curves were constructed by Kaplan–Meier analysis, and the differences between groups were tested by log-rank test. For non-parametrical statistical hypothesis, Wilcoxon rank-sum test was run for two categories, and Kruskal-Wallis test was applied for three or more categories. The “limma” package was used for normalization and differentiation analysis. The R software (Version 4.0.1) laid the basis for all statistical analyses. All statistical tests were double-tailed, and statistical significance was set by P <0.05.

## Results

### Landscape of Genome Mutation in SCC

**Figure 1:**
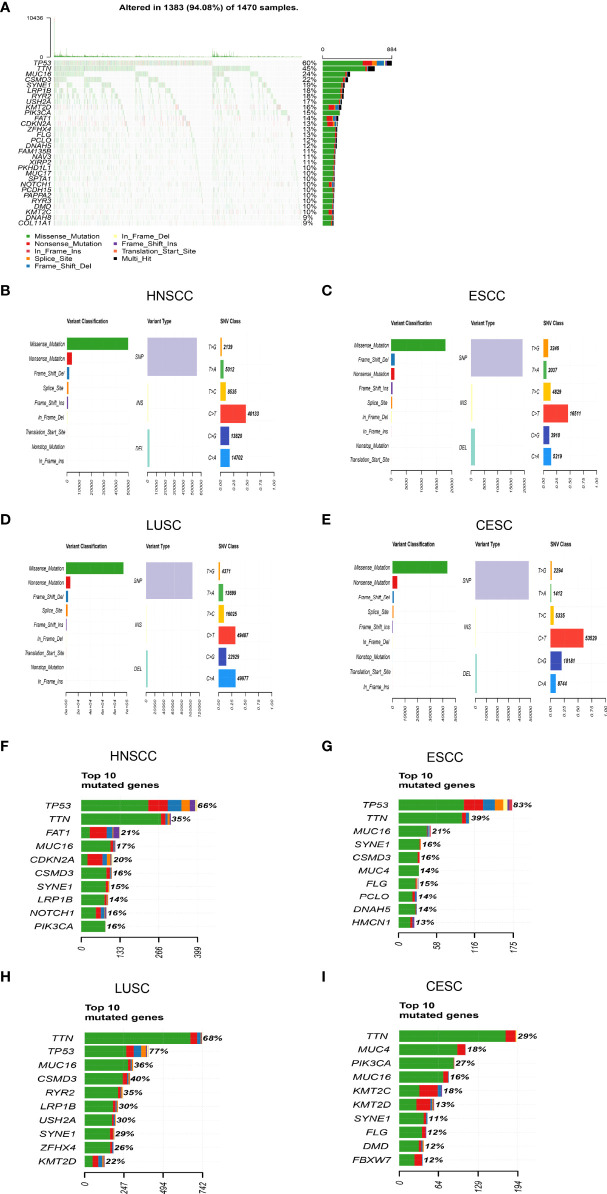
Summary of mutational landscape in SCC patients from TCGA database **(A)**Mutation information in each SCC sample was shown in the waterfall plot, in which various colors representing different mutation types were annotated at the bottom. **(B–E)** Missense mutation was the most common variant classification, and SNP was the most dominant mutation type in SCC. The C > T transition was the most frequent SNV in HNSCC, ESCC and CESC. The transition of C>A was more common in LUSC. **(F–I)** The top10 mutated genes were displayed respectively in different SCCs.

Somatic mutation data of 1470 SCC samples were downloaded from the Cancer Genome Atlas database, including sample name, chromosome where the mutation occurred, starting and ending location of mutation, mutation classification, mutation type, etc. In the waterfall plot, 1383 (94.08%) SCC patients occurred somatic mutations, with mutation types represented by different color-coded annotations (**Figure 1A**). The following findings were consistent in HNSCC, ESCC, LUSC and CESC. Missense mutation was the most common variant classification, followed by nonsense mutation and frameshift deletion. In addition, single nucleotide polymorphism (SNP) was the most dominant mutation type. However, the C > T transition was the most frequent single nucleotide variants (SNV) in HNSCC, ESCC and CESC. The transition of C>A was more common in LUSC (**Figures 1B–E**). The top10 mutated genes were displayed by horizontal histogram (**Figures 1F–I**). It can be seen that the top10 mutated genes in HNSCC included TP53, TTN, FAT1, CDKN2A, MUC16, C SMD3, NOTCH1, PIK3CA, SYNE1 and LRP1B. The top10 mutated genes in ESCC were TP53, TTN, MUC16, SYNE1, CSMD3, FLG, MUC4, PCLO, DNAH5 and HMCN1. The top10 mutated genes in LUSC were TP53, TTN, CSMD3, MUC16, RYR2, LRP1B, USH2A, SYNE1, ZFHX4 and KMT2D. The top10 mutated genes in CESC were TTN, PIK3CA, MUC4, KMT2C, MUC16, KMT2D, FLG, DMD, FBXW7 and SYNE1. The sequences of mutated genes in horizontal histogram were based on the total number of mutations that had occurred. The proportions of the number of samples with genetic mutations to the total number of samples were expressed as percentages. Consequently, the above two orders were slightly different.

### TMB Correlated With Prognosis and Clinical Characteristics

**Figure 2:**
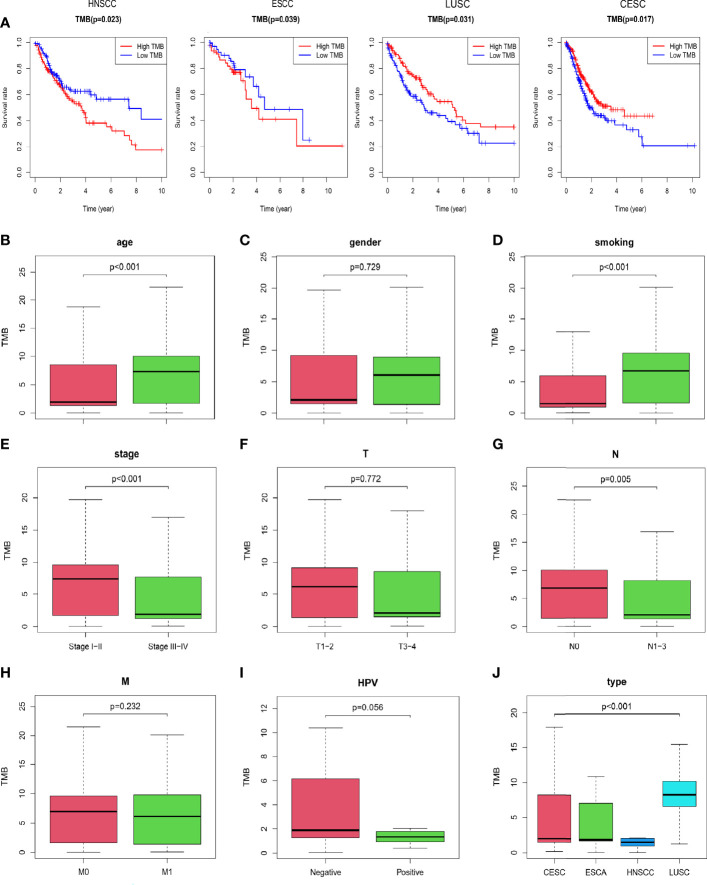
Prognosis of TMB and association with clinical characteristics **(A)** Patients with low TMB had better prognosis In HNSCC and ESCC. Patients with high TMB had better prognosis in LUSC and CESC. **(B, D, E, G)** Patients with older age, smoking history, lower pathological stage, and no lymphatic invasion had higher TMB. **(C, F, H)** No significant correlations were observed between TMB and gender, AJCC-T stage, and AJCC-M stage. **(I)** There was no significant correlation between TMB and HPV infection. **(J)** TMB was significantly different among the four types of SCCs, with the highest TMB in LUSC.

Clinical data of SCCs downloaded from the Cancer Genome Atlas was shown in detail (**Table 1**). Kaplan-Meier survival curve showed that TMB was associated with prognosis. However, TMB was not consistent with prognosis in different SCCs (**Figure 2A**). In HNSCC and ESCC, patients with low TMB had better prognosis (p=0.023, p=0.039). But, patients with high TMB had better prognosis in LUSC and CESC (p=0.031, p=0.017). In addition, the relationship between TMB and clinical features had also been described in SCCs. The results indicated that patients with older age (p<0.001), smoking history (p<0.001), lower pathological stages (p<0.001), and no lymphatic invasion (p=0.005) generally had higher TMB (**Figures 2B, D, E, G**). However, no significant correlations were observed between TMB and gender, AJCC-T stage, and AJCC-M stage (**Figures 2C, F, H**). As we all know, HPV infection is a risk factor for HNSCC and CESC. We also analyzed the relationship between HPV status and TMB in HNSCC and CESC. But the result showed no significant association between TMB and HPV infection (**Figure 2I**). Furthermore, TMB differed considerably among the four types of SCCs, with the highest TMB in LUSC (**Figure 2J**, p<0.001).

**Table 1:** Clinical characteristics of 1326 patients with SCC from TCGA database.

Variables	Number (%)
Vital status	
Alive	812 (61.24%)
Dead	514 (38.76%)
Age, y	60.74 ± 13.13
Gender	
Female	525 (39.59%)
Male	801 (60.41%)
HPV status	
Positive	65 (4.90%)
Negative	88 (6.63%)
Unknow	1173 (88.6%)
Smoking history	
Yes	788 (59.43%)
No	339 (25.56%)
Unknow	199 (15.01%)
Pathological stage	
Stage I & II	651 (49.10%)
Stage III & IV	461 (34.77%)
Unknow	214 (16.14%)
AJCC-T	
T1&T2	793 (59.80%)
T3&T4	417 (31.45%)
TX	116 (8.75%)
AJCC-N	
N0	792 (59.73%)
N1-3	489 (36.88%)
NX	45 (3.39%)
AJCC-M	
M0	823 (62.07%)
M1	101 (7.61%)
MX	402 (30.32%)

### Differentially Expressed Genes Between Two TMB Groups and Functional Pathway Analysis

**Figure 3:**
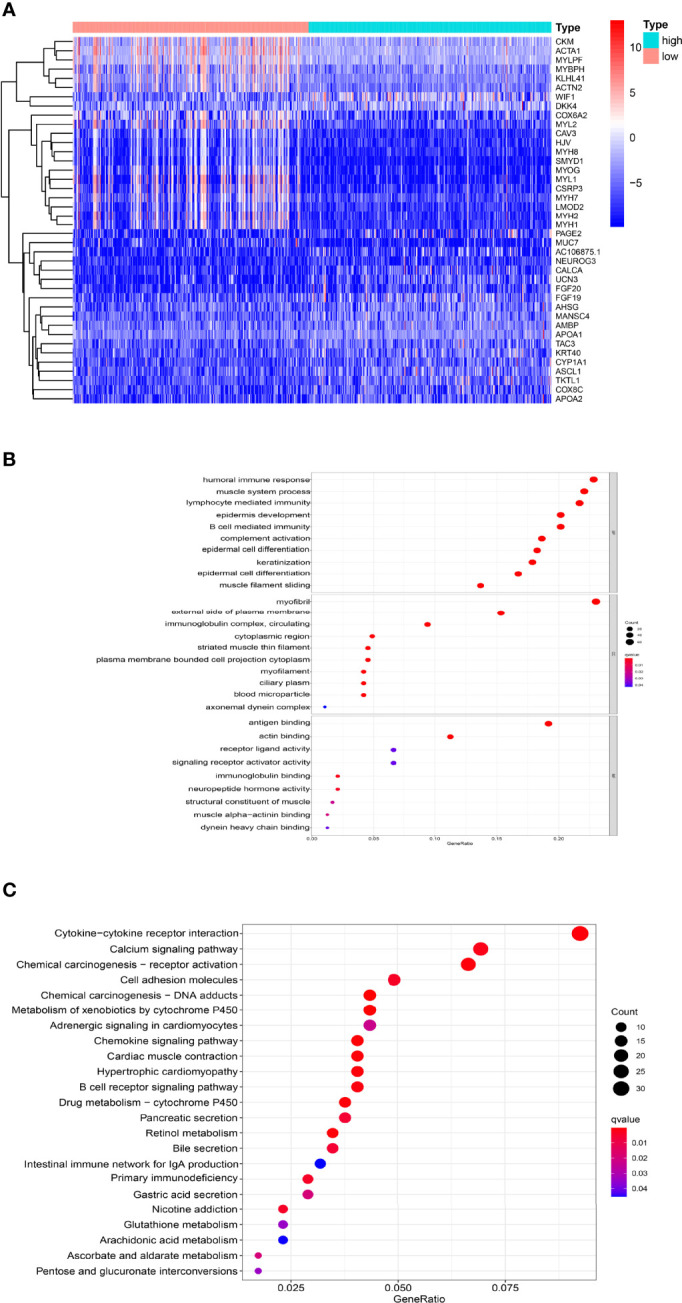
Transcriptome analysis of high TMB and low TMB groups **(A)** The heatmap showed the top 40 DEGs between two TMB groups. **(B)** GO analysis revealed that DEGs were involved in muscle system process, the activity of epidermal cells and immune-related function. **(C)** KEGG pathway analysis of DEGs was found in immune signal mediation, cytochrome P450, cytokine regulation signaling pathways.

1282 DEGs with FDR <0.05 and∣|log (fold change) > 1| were screened by “limma” package, including 876 upregulated genes and 406 downregulated genes in high-TMB group. The expression of the top 40 DEGs in the two TMB groups was shown by heatmap (**Figure 3A**). In GO enrichment analysis, it was found that DEGs were mainly involved in muscle system process, the activity of epidermal cells and immune-related functions (**Figure 3B**). According to KEGG pathway analysis, DEGs was found in immune signal mediation, cytochrome P450, cytokine regulation and other signaling pathways (**Figure 3C**).

### Comparison of Immune Cell Infiltration

**Figure 4:**
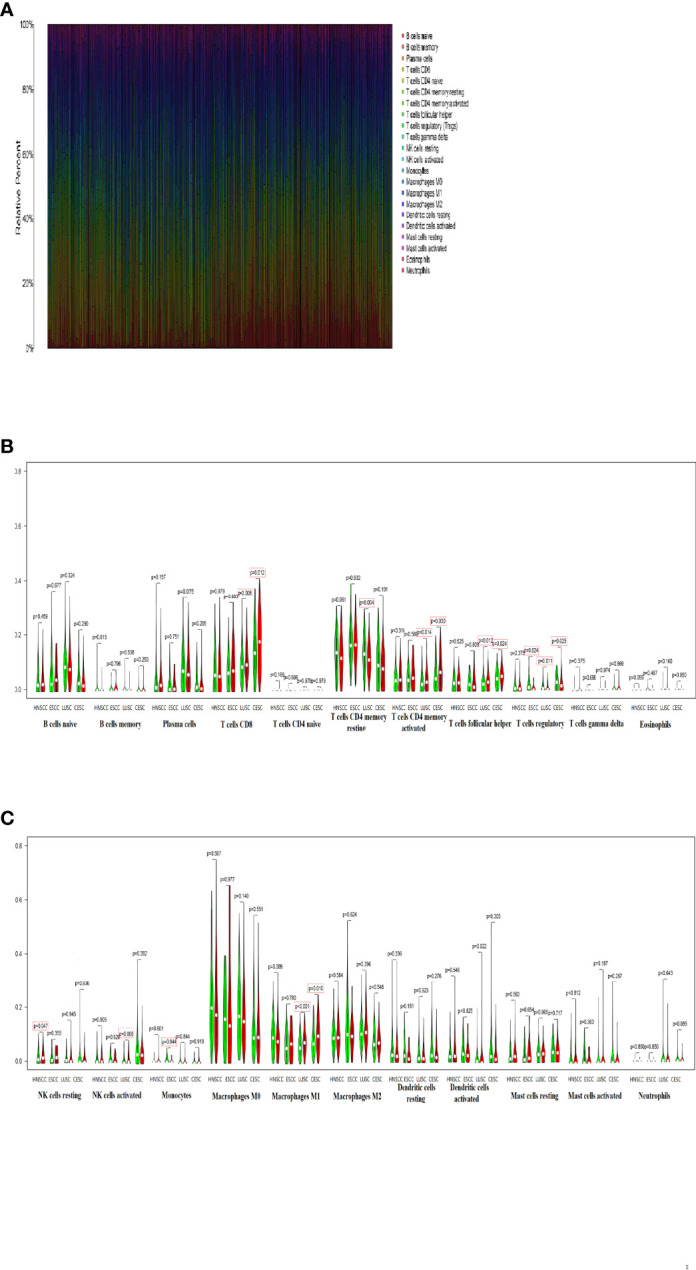
Comparison of immune cell infiltrations in different types of SCCs **(A)** 22 immune cells proportion in each SCC sample were shown in barplot. **(B, C)** Immune cell infiltrations were different between two TMB groups in HNSCC, ESCC, LUSC and CESC.

CIBERSORT algorithm was used to estimate the relative proportion of 22 immune cells represented by various colors in each SCC sample (**Figure 4A**). Then, we compared the differences of immune cell infiltrations between low-TMB group and high-TMB group in these four types of SCCs. In the violin plot, low-TMB group was represented in green, while high-TMB group in red (**Figures 4B, C**). It was found that high-TMB group had more CD8 T cells in LUSC and CESC (p=0.008, p=0.012), less CD4 memory resting T cells in LUSC (p=0.004), more CD4 memory activated T cells in LUSC and CESC (p=0.014, p=0.030), more follicular helper T cells in LUSC (p=0.012), less regulatory T cells in ESCC, LUSC and CESC (p=0.024, p=0.011, p=0.025), more resting NK cells in HNSCC (p=0.047), more activated NK cells in LUSC (p=0.006), less monocytes in ESCC (p=0.044), more macrophages M1 in LUSC and CESC (p<0.001, p=0.010).

### Immune-Related DEGs and Prognostic Model

**Figure 5:**
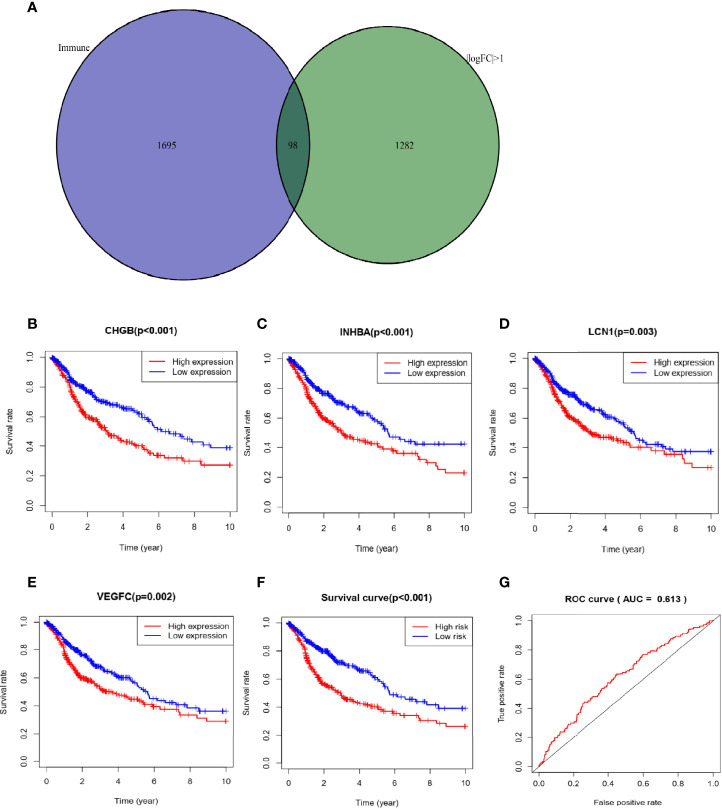
Construction of prognostic model for SCC **(A)** Immune-related DEGs was shown in Venn plot. **(B–E)** High expression of hub genes was associated with poor prognosis. **(F)** The high-risk group had worse prognosis. **(G)** The area under the curve (AUC) was 0.613.

1695 immune-related genes were downloaded from the immune omics database. Then, we identified 98 immune-related DEGs that overlapped between immune-related genes and DEGs through “VennDiagram” package (**Figure 5A**). Then four hub genes (CHGB, INHBA, LCN1 and VEGFC) related to prognosis were selected from 98 immune-related genes *via* univariate and multivariate cox analysis. The survival curve showed that high expression of these four genes was associated with poor prognosis (**Figures 5B–E**). To explore the significance of hub genes in assessing the prognosis of SCC patients, the following formula was used to calculate risk score for each patient: risk score= -0.0243×expression of LCN1+0.0029×expression of CHGB+0.0041×expression of INHBA+0.0121×expression of VEGFC. We classified patients into high and low risk groups based on the median risk score. The results showed that the high-risk group had worse prognosis (**Figure 5F**, p<0.001). The area under the curve (AUC) was 0.613 (**Figure 5G**), which had certain predictive value.

### Analysis Based on TIMER Database

**Figure 6:**
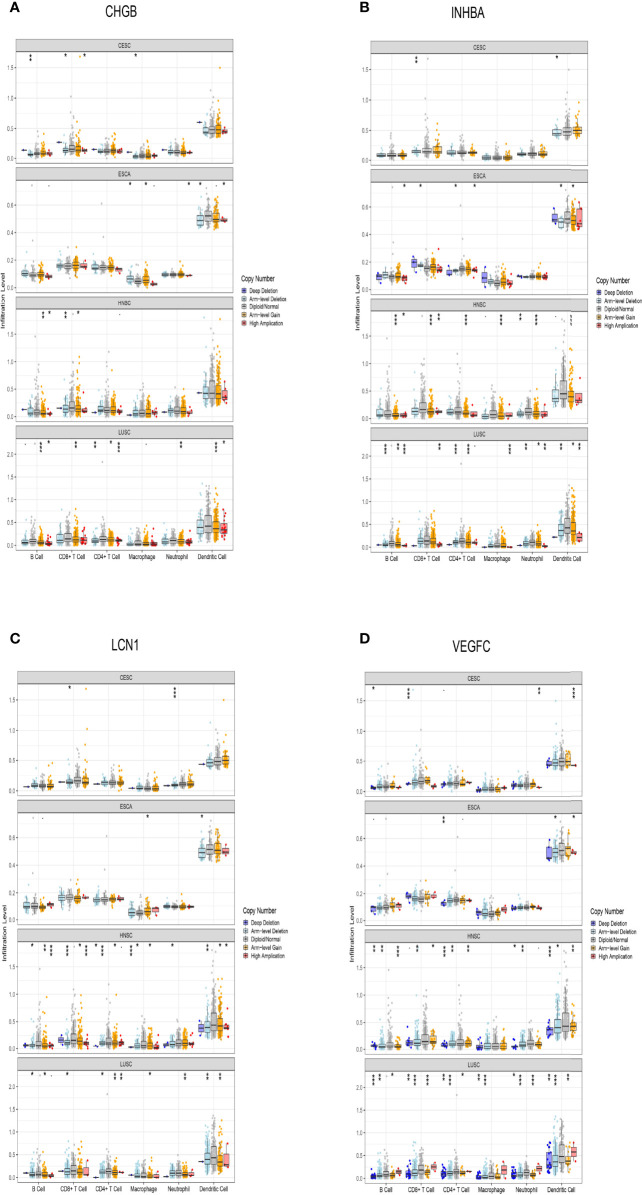
Association between somatic copy number alteration (CNA) of prognosis-related hub genes and immune cell infiltration levels. **(A–D)** CNA of hub genes could reduce immune cell infiltration. * means p< 0.05, ** p< 0.01, *** p< 0.001.

We explored the relationship between copy number alteration (CNA) of prognosis-related hub genes and immune cell infiltration *via* TIMER database “SCNA” module. Comparing with diploid/normal expression of hub genes, we found that CNA of hub genes could reduce immune cell infiltration, including B cells, CD8+ T cells, CD4+ T cells, macrophages, neutrophils, and dendritic cells (**Figures 6A–D**).

## Discussion

Immunotherapy subverts the previous concept of anti-tumor therapy, which shifts from relying on the outside world to relying on the own immune system to kill cancer cells. However, the clinical application of immunotherapy still has significant complexity and uncertainty, and still faces some challenges related to efficacy and safety. Although many studies are currently exploring the mechanisms of antitumor immunotherapy, the understanding of biomarkers that predicts immunotherapy sensitivity and drug resistance is still preliminary. PD-L1 expression is one of the most studied predictive markers, and several anti-tumor immunotherapy drugs based on PD-L1 protein expression have been approved for marketing (13). However, PD-L1 is not always a perfect biomarker due to the heterogeneity and instability inherent in tumors (14). Therefore, several candidate biomarkers have been extensively studied, including DNA mismatch repair defects (dMMR), microsatellite instability (MSI), tumor-infiltrating lymphocytes (TILs), tumor mutation burden (TMB), and so on.

A number of studies have shown that TMB is associated with immunotherapy efficacy in a variety of tumors. The anti-tumor effects of the immune system depend on its effective recognition of antigen. A few somatic mutations in tumors can produce neoantigens that can be recognized and targeted by the immune system. Importantly, not all mutations produce neoantigens. In fact, only a few mutations can produce neoantigen-containing peptides. These peptides are processed by antigen-processing mechanisms and loaded onto the major histocompatibility complex (MHC), and even fewer are recognized by T cells (15–17). Therefore, the prevailing view is that the more mutations detected, the more probable it is to generate neoantigens, and the more likely these neoantigens are to be immunogenic and trigger T-cell responses. TMB was first identified as a biomarker for immune checkpoint inhibitors in melanoma (18). Recently, the Food and Drug Administration (FDA) approved pembrolizumab for adult and pediatric patients with TMB > 10Mut/Mb. This approval was based on efficacy data from 10 refractory solid tumor cohorts participated in a multicenter, non-randomized, open-label study KEYNOTE-158 (19). The number of somatic mutations varied significantly across many tumor types, with melanoma having the highest number of mutations, followed by non-small cell lung cancer and other squamous cancers, and leukemia and certain childhood cancers having the lowest number of mutations (20, 21). In addition, the predictive value of TMB for overall survival was inconsistent among different cancers. There are some limitations to the potential use of TMB in practice, making this approval highly controversial (22). It can be seen that TMB as a possible universal biomarker of pan-cancer has certain advantages, but also has inherent limitations.

Tumor development is closely related to genetic mutations of key molecules. Most of the mutations found in tumors are already present in normal tissue, so the accumulation and combination of these mutations may be more important than their occurrence alone (23). The types and frequency of mutations also vary widely among different typs of tumors. This study systematically analyzed mutation profiles in four common squamous cell carcinomas, which has certain clinical significance for precision immunotherapy.

First, we focused on analyzing the differences in the mutational status of four SCCs. Single nucleotide mutations are caused by the substitution of a single base. The changes are related to prediction of disease, response to drugs and tumor pathogenesis. Mutations are usually enriched in a specific local sequence situation. For instance, ultraviolet induced pyrimidine dimers, whose faulty repair results in C>T mutations of at CpC or TpC dinucleotides. The mutations associated with smoking were mainly C > A mutations (24). Certain genetic mutations appear to be more frequent or potentially specific in specific squamous cell carcinomas. The most common mutated genes were TP53 and TTN in HNSCC, ESCC and LUSC. Differently, the most common in CESC were TTN and PIK3CA. As one of the most important tumor suppressor genes, P53 plays a critical role in tumor development because it controls cell growth, apoptosis and regulates angiogenesis. Missense mutations of TP53 lead to the expression of a conformationally altered stable protein that has negative activity against wild-type P53 and has also acquired functional carcinogenic activity. Thus, mutation of only one TP53 allele may result in a significant oncogenic phenotype (25, 26). TTN, the second most mutated gene in solid tumors, is the gene encoding the sarcomere protein, which plays a key structural, developmental and regulatory role in heart and skeletal muscle (27). TTN mutation predicts higher TMB and correlates with the response rate to immune checkpoint blockade (28). It is also found that the frequency of gene mutation is positively correlated with the length of exon. TTN is the gene with the longest exon length in the whole genome. As the second longest gene in the genome, MUC16 has a high mutation frequency, which is associated with significant tumor mutation load. This result also supports the correlation between higher mutational load and mutational status of genes with long exons (29). Previous studies have found that patients with TTN/TP53 dual mutations have better benefits in OS and DFS compared with patients with TTNWT/TP53MT status, suggesting that TTN and TP53 mutations may have synergistic effects in LUSC (). PIK3CA is the most commonly mutated gene in human papillomavirus (HPV) associated squamous cell carcinoma and is an important factor in predicting the prognosis of cervical cancer patients (31–33).

Moreover, we analyzed the correlation of TMB with prognosis and clinical traits. In our study, high TMB was associated with smoking and HPV negative. Some mutational processes can lead to high TMB, such as POLE/POLD1 mutation, mismatch repair deficiency, UV light, tobacco smoking, AID/APOBEC activation. Thus, cancers associated with chronic mutagen exposure, such as lung cancer (tobacco) and melanoma (UV), show higher TMB (25). This may be the reason why LUSC has the highest TMB in these four types of SCCs. Smoking-related mutations have been found to be associated with responses to checkpoint blockade and thus may underlie some tumor responses to PD-1 pathway blockade (10). However, TMB may have different effects on tumor immunity depending on anatomic location. In HNSCC, smoking is mainly immunosuppressive. In LUSC, it’s more conducive to inflammatory response (24). After HPV infection, the E7 oncoprotein was found to cause centrosomal abnormalities that disrupt mitosis and increase the risk of chromosome misalignment and aneuploidy, while chromosome instability may lead to increased genetic mutations. In theory, HPV-positive patients should have higher TMB values (34). We also found that older patients had higher TMB. This has also been confirmed in previous studies. TMB increases significantly with age, with a 2.4-fold difference between 10 and 90 years old (35).

In addition, it was found that TMB related DEGs were enriched in the regulation of immune system and muscular system through GO and KEGG pathway analysis. We also identified four immune-related DEGs that were strongly associated with poor prognosis, including: CHGB, INHBA, LCN1 and VEGFC. CHGB was first identified in pheochromocytoma and encodes proteins that are mainly expressed in endocrine cells and neurons (36). Abnormal expression of CHGB gene has been reported in many tumor types, and its upregulated expression is highly correlated with metastasis (37, 38). INHBA, encoding a member of the TGF-beta superfamily of proteins, has been shown to be associated with poor prognosis in a variety of solid tumors^39^). The molecular mechanism and tumor-promoting function of INHBA remain unclear. Currently, most hypotheses focus on metastasis. Wamsley et al. suggested that activins were necessary to maintain a cancer stem cell-like phenotype and contribute to metastasis of NSCLC (40). 41 also confirmed this viewpoint in HNSCC (41). LCN1 (lipocalin-1), known as tear lipocalin, is mainly expressed in secretory glands and tissues (42). It has been reported that LCN1 overexpression is an independent predictor of poor prognosis in breast cancer (43). However, few studies have investigated its expression level in other malignant tumors. Vascular endothelial growth factor C (VEGFC), an activator of lymphangiogenesis, plays an important role in promoting lymph node metastasis and tumor progression (44).

Based on the plotted survival curve, we found that LUSC and CESC patients with high TMB were significantly associated with better survival, while HNSCC and ESCC patients with high TMB had poor prognosis. The mechanism behind this association may lie in the significant differences in immune cell invasion density and immune activity between low and high TMB subtypes of these cancers (45). We analyzed the association between TMB and immune cell infiltration in squamous cell carcinoma and found that these associations were often related to the type of cancer. It can be seen in the violin plot of immune cell infiltration, high-TMB group had more CD8 T cells and less regulatory T cells in LUSC and CESC. McGrail et al. analyzed somatic mutation data from more than 10,000 patients in the TCGA database and determined the association between predicted neoantigen load and CD8 T cells. They found that in cancers where CD8 T cell levels were positively correlated with neoantigen load, such as melanoma, lung cancer, and bladder cancer, high-TMB tumors had significantly higher ORR than low-TMB tumors (21). A retrospective study also showed that increased CD8+ T cell infiltration and increased CD8+ T cell/regulatory T cell ratio were positively associated with ICB treatment response (46).

Macrophages are important immune cells in tumor microenvironment and can be polarized into subtypes with different functions in different microenvironments, including M1 and M2 macrophages. M1 macrophages secrete cytokines such as TNF-α, which have anti-tumor, anti-angiogenesis and activation of adaptive immunity. Tumor-associated macrophages (TAMs) is an important regulator of tumorgenesis, usually manifested as M2 subtype. It inhibits Th1 immunity by promoting tumor angiogenesis and invasion and is associated with poor prognosis (47). In this study, we found that the group with high TMB had higher macrophage M1 infiltration in LUSC and CESC. This may also be one of the reasons why high TMB group has better prognosis in LUSC and CESC. In clinical trials, it could be used to stratize patients and assign the most appropriate treatment according to the type of target cell, thus increasing the chances of overall success.

Inevitably, there were also a few limitations in the investigation. This study was a retrospective analysis using public database and the results have not been validated in prospective clinical trials. Therefore, relevant conclusions need to be further studied. Although further validation is required, these results may provide new insights into the determinants of immunotherapy response to SCCs.

## Conclusions

Based on TCGA database, this study systematically elaborated the effect of TMB on the prognosis and the relationship between TMB and immune cell infiltration of SCCs. We found that TMB has different effects on prognosis in SCCs at different anatomical sites, which may be related to the difference in immune cell infiltration. In addition, we identified 4 hub genes associated with prognosis and constructed a risk prognosis model. However further studies are needed to verify the clinical application of this prognostic model. Overall, new insights can be gained by regarding different SCCs as a whole.

## Data Availability Statement

The original contributions presented in the study are included in the article/supplementary material. Further inquiries can be directed to the corresponding authors.

## Author Contributions

YC and JW contributed to the design of the study. WT and TW collected and analyzed the TCGA data. WT was the major contributor in writing the manuscript. YC supervised the study. All authors read and approved the final version of the manuscript.

## Funding

This work was supported by the Special fund for Taishan Scholar Project (NO. ts20190973).

## Conflict of Interest

The authors declare that the research was conducted in the absence of any commercial or financial relationships that could be construed as a potential conflict of interest.

## Publisher’s Note

All claims expressed in this article are solely those of the authors and do not necessarily represent those of their affiliated organizations, or those of the publisher, the editors and the reviewers. Any product that may be evaluated in this article, or claim that may be made by its manufacturer, is not guaranteed or endorsed by the publisher.
